# The Outcomes of Traumatic Acute Subdural Hematoma in a Tertiary Center in Abuja, Nigeria

**DOI:** 10.7759/cureus.20016

**Published:** 2021-11-29

**Authors:** Kenechukwu K Igbokwe, Obinna M Ayogu, Daniel E Onobun, Edidiong A Essiet, Ugochukwu C Ugwuanyi

**Affiliations:** 1 Neurosurgery, Wellington Neurosurgery Centre, Abuja, NGA; 2 Neurosurgery, National Hospital Abuja, Abuja, NGA

**Keywords:** surgical outcome, functional outcome, time from injury to surgery, brain trauma injury, glasgow coma scale, acute subdural hematoma

## Abstract

Background

Acute traumatic subdural hematoma is life-threatening and is associated with high unfavorable outcomes in developing countries.

Objective

We aim to identify factors contributing to outcomes after severe traumatic brain injury (TBI) due to acute subdural hematoma (SDH) in patients admitted to National Hospital Abuja, Nigeria.

Methods

This was a retrospective review of 34 patients who consecutively underwent neurosurgery for acute SDH over five years (from January 2015 to December 2019). Demographic data, clinical characteristics, and the time intervals from injury to surgery were investigated to determine the interactions between all these factors and outcome. Outcome was graded according to the Glasgow outcome scale at the three-month follow-up.

Results

Out of 34 patients who had surgical evacuation for traumatic acute subdural hematoma, 15 patients died (44.1%). A significant correlation was identified between outcome and the Glasgow coma scale score at admission. No significant correlation was seen between the outcome and the age, gender and the time from injury to surgery (chi^2^ test, p>0.05).

Conclusion

The rate of unfavorable outcomes in acute subdural hematoma is high. The Glasgow coma score at admission is an important predictor for outcome in traumatic acute subdural hematoma.

## Introduction

Traumatic brain injury (TBI) is associated with morbidity, deformity, and mortality, leaving its victim with varying degrees of dependency, emotional and economic burden. It occurs as a result of insult to the brain within the cranial vault. Several studies have shown that it is a major cause of death in Nigeria affecting mostly men [1-4]. Traumatic acute subdural hematoma (ASDH) is one of the most devastating types of traumatic brain injury, with a mortality rate ranging from 30% to 70% [5-7]. The advent of computerized tomography (CT) has increased the ability of clinicians to accurately characterize these lesions on imaging and has brought about a significant decline in mortality rates worldwide. However, mortality rates remain high in developing countries [8]. Most cases of subdural hematomas (SDH) were unilateral and involved the parietal lobe [9].

Several factors have been implicated in the prognosis of ASDH. These include Glasgow coma scale (GCS) score, pupillary abnormalities, systemic blood pressure, respiratory rate, glycaemic status, length of hospital stay, hypoxia, presence or absence of a subarachnoid bleed, and intraventricular hemorrhage [6,7,9,10,11]. Prompt surgical intervention has been shown to reduce the mortality rate of ASDH if the subdural hematoma was removed within four hours after injury [5].

Thus, we evaluated the data of traumatic ASDH patients who were surgically treated from January 2015 to December 2019 in National Hospital Abuja, Nigeria. In this study, the preoperative variables were correlated with outcomes at three months post-discharge. We aimed to determine the degree to which the proposed variables impact the outcome of patients with traumatic ASDH.

## Materials and methods

This retrospective study was conducted in the trauma center of National Hospital Abuja, the largest neurotrauma center in north-central Nigeria. Thirty-four patient records of patients admitted for acute subdural hematoma between January 2015 and December 2019 were retrieved and reviewed. We reviewed the final diagnosis of all patients and excluded patients with nontraumatic acute subdural hematoma. The following factors from the groups were analyzed: demographic data (age and sex), the time between the trauma and surgery, GCS score at admission, and Glasgow outcome scale (GOS) score at the third-month follow-up were investigated. Factors that affected the outcomes were determined. 

The Glasgow coma scale (GCS) score was used to classify the severity of the injuries. Patients with GCS of 13-15, 9-12, and ≤8 were classified as mild, moderate, and severe injury respectively. The outcome was measured using GOS at the third-month post-discharge. The GOS 1 indicated mortality and GOS 2-4 indicated a residual disability whereas GOS 5 indicated good functional recovery. The GOS 2 indicated vegetative state, GOS 3 indicated severe disability, GOS 4 implied moderate disability. Only GOS 5 was used to describe a favorable outcome while GOS scores 1-4 were regarded as unfavorable outcomes. This scale was chosen because of its established validity, interobserver invariability, and its wide adoption as a standard means of describing outcomes in head injury.

Data were collected from previously prepared patient information sheets. Statistical analyses were performed using the Statistical Package for social sciences (SPSS) version 28.0 (IBM Corp., Armonk, NY, US). Mean ± standard deviation (SD) was employed for numerical variables like age; whereas frequencies and percentages were computed for categorical variables like gender, the time between injury and surgery, GCS, GOS, and outcome. The stratification was done with regards to age, gender, the time between injury and surgery. The time between injury and surgery was described as the total delay. A Chi-square test was used to observe the relationship between different variable factors and outcome at three months post-discharge. Any association having a probability value (p-value) of <0.05 was considered statistically significant.

## Results

Thirty-four patients were operated on for traumatic acute subdural hematoma at National Hospital Abuja, Nigeria. Of the total of 34 cases, 30 were males, and four were females, with a male to female ratio of 7.5:1. The mean age was 36.26 years (±12.676), with a range from nine years to 60 years. There was an uneven age distribution as most patients (58.8%) were in the 25 to 45 years age category. Five of the eight patients (62.5%) with good functional outcomes were between the ages of 14 to 25 years (Table 1).

**Table 1:** Age and gender tabulated against the outcome

Clinical variables	No of patients (n=34)	Mortality (n=15)	Residual disability (n=10)	Functional recovery (n=9)	p-value
Gender					
Male	30	13	9	8	0.989
Female	4	2	1	1	
Age (years)					
0-13	1	0	1	0	0.291
14-25	8	2	1	5	
26-45	18	8	7	3	
46-60	7	5	1	1	
≥61	0	0	0	0	

**Figure 1:**
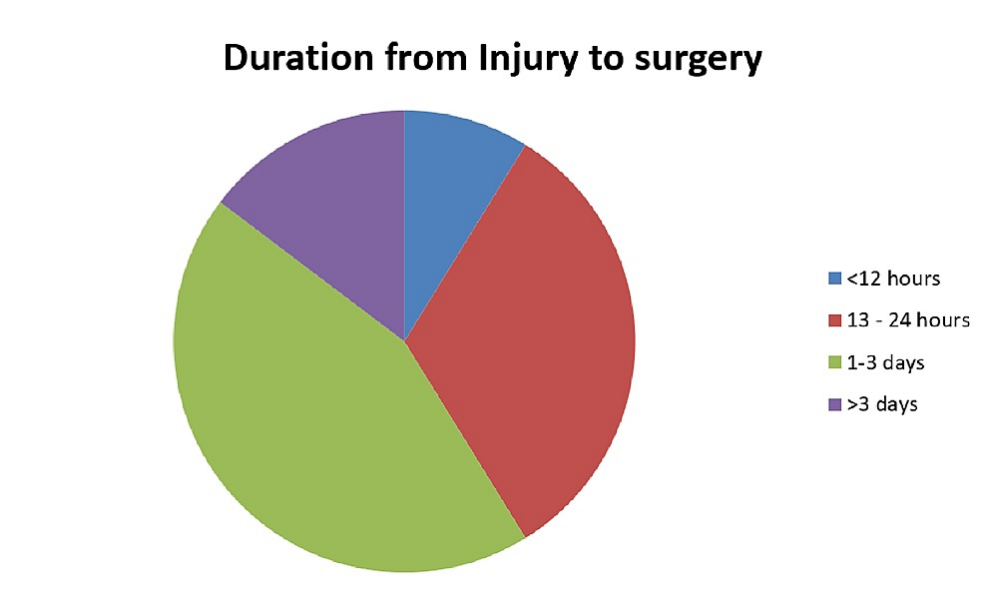
Duration from time of injury to surgery

The time from injury to surgery, also referred to as the total delay, was examined. The average total delay was 49.8 hours (±75.3 hours) with 41.2% being operated in the first 24 hours post-injury, 44.1% within one to three days, 8.8% from four to seven days post-incident, and 5.9% being operated more than one-week post-injury (Figure 1).

The injury severity at presentation assessed using the GCS showed 17.6% (six of 34) of patients presented with mild injury, 40.6% (13 of 34) of patients presented with moderate injury while severe brain injury was seen in (GCS≤8) 46.9%. An overall 44.1% (15 of 34) mortality was recorded, with most deaths in the severely injured patients (11 of 15) deaths. There is an uneven distribution of mortality in these categories. One death (7.7%) was recorded for the moderately injured group and three patients died in the mildly injured group, two had a residual disability and one had a full recovery. Good functional outcome was recorded in 55.9% (seven of 13) of these patients with moderate injury. Only one of the patients who presented with GCS of 13-15 had a favorable outcome. Around 50.0% of patients (three of six) with mild injury, died (Table 2).

**Table 2:** Glasgow coma score tabulated with surgical outcome

Glasgow coma scale (GCS) score	Mortality (n=15)	Residual disability (n=10)	Functional recovery (n=9)
Severe (GCS ≤ 8)	11	3	1
Moderate (GCS 9-12)	1	5	7
Mild (GCS 13-15)	3	2	1

## Discussion

Our findings demonstrate the outcome of ASDH in a major referral center in northern Nigeria. Acute subdural hematoma (ASDH) develops in approximately a third of brain injuries due to severe head trauma [8] and presents as a life-threatening condition in the neurosurgical emergency. Its mortality rate can be as high as 70% [5]. Of our 34 patients, the mortality was 44.1%, which is comparable to previously reported high mortality rates [5-7]. This mortality rate is significantly higher in comparison to traumatic extradural hematomas which have mortality rates approaching zero with the advent of CT imaging [12,13].

The patients in this study were predominantly male, with a male to female ratio of 7.5:1. This corroborates the outcome of two previous studies which reported male preponderance in acute subdural hematoma [8,10]. This ratio is a reflection of our social culture where females are not exposed to unskilled external work and other work that involves a lot of travel. In this study, the proportion of unfavorable outcomes in both genders was nearly even (M: F 73.3%: 75%). The mortality rate, however, was lower in men (M: F 38.2%: 50%). This difference failed to achieve statistical significance as a predictor of outcome (p=0.989). 

Increasing age has been associated with higher mortality from traumatic brain injury [1,6]. In our study, we observed a higher mortality rate (52.5%, n=13) in patients aged 26 to 60 (n=25), compared to the mortality rate (22.2%, n=2) in patients between the ages of zero to 25 (n=9). The mortality rate in older patients is twice the rate in younger patients. Some of this increased mortality may be explained by intrinsic clinical properties of the older patients who may have pre-existing co-morbidities that may worsen their clinical prognosis. Despite this finding, a statistically significant relationship between age and outcome was not established in this study (p=0.291). This is in contrast to other studies by Kulesza et al., and Leitgeb et al., that reported a statistically significant relationship between age and outcome in acute subdural hematoma in their study of 100 and 218 patients, respectively [10,14]. It is possible that the smaller sample size had a role to play in the statistical significance of this finding.

In this study, all patients were operated on as soon as possible after indications for surgical evacuation of subdural hematoma were identified. Hematoma evacuation was dependent on the GCS score, hematoma thickness, parenchymal edema of the patient, and other factors. The surgical evacuation was achieved by decompressive craniectomy or craniotomy. Decompressive craniectomy was used in patients with lower GCS and severe edema to provide enough space for brain relaxation and to prevent herniation [7,8]. Bullock et al. described the indications for surgical evacuation in ASDH, these include (i) thickness greater than 10mm or midline shift greater than 5mm on CT and (ii) GCS less than 9 plus SDH less than 10mm or midline shift less than 5mm if the GCS score decreased at any point in the delays between injury and hospital admission [15].

In Nigeria, the neurosurgical service is still evolving and as such, there tend to be unacceptable delays before appropriate referral to a competent neurosurgical center is completed [13]. In the present study, the time interval from injury to surgery was an average of 49.8 hours (±75.3 hours). The majority of the patients had surgical evacuation between one to three days from injury. This subset of patients had a favorable outcome of 40.0% (six of 15 patients). Only three of 14 (21.4%) patients had a favorable outcome in the group that had surgical evacuation of ASDH in the first 24 hours (41%, n=14). None of the patients who were operated on after four days (14.7%, n=5) had a favorable outcome as mortality was recorded in 60% (three of five) and 40% (two of five) had a residual disability at the third-month follow-up. This finding contrasts with a study that found a significant association between the early evacuation of hematoma within two hours and favorable surgical outcomes [5]. A reduction in mortality rate from 90% to 30% was reported if surgical evacuation for hematoma is done within four hours [16]. In our study, we did not identify any significance between the time interval from injury to surgery and surgical outcome (p = 0.483). Several studies have failed to identify the effect of time to surgery on surgical outcomes [6,17,18]. This finding can be explained by a theory that suggests a consequent increase in brain edema after early decompressive craniectomy due to changes in interstitial fluid pressure gradients. Another theory suggests cerebral infarction with hemorrhagic transformation after early decompressive surgery [17]. Despite this, several studies recommend early surgical decompression of acute subdural hematoma [13,17].

Patients with ASDH frequently presented with altered states of consciousness. The GCS is a practical classification method that directly reflects brain damage, reflects clinical status, and provides information on survival during follow-up. The GCS at presentation has been regarded as one of the most important predictors of surgical outcome in acute subdural hematoma [5,7,8,10,11]. Lower GCS scores were associated with a higher incidence of mortality and residual disability. This assertion corroborates with our study findings of a higher mortality incidence (73.3%) in the group of patients with GCS 3-8. Statistical analysis revealed a significant relationship between the GCS and the outcome at three months post-discharge (p=0.029).

The overall mortality rate from this study was high (44.1%) but is similar to some other studies [10,14]. This finding could be explained by several factors not identified in this study. In patients presenting after 72 hours, mortality was higher (60%, n=5, p=0.483) than in those presenting earlier and had no favorable outcome. Among the patients that died, 78% (11 of 14) had a severe injury (GCS <9) at presentation. However, 12 of the 13 patients admitted with a GCS of 9-12 in our study survived, and seven of them had a favorable outcome.

## Conclusions

The rate of unfavorable outcomes in acute subdural hematoma is high in comparison to acute epidural hematoma. The Glasgow coma score at admission is an important predictor for outcome in traumatic acute subdural hematoma. There was no significant relationship between the outcomes and the age, gender, and time from injury to surgery. Despite this finding, it is reasonable to perform a timely surgical decompression of acute subdural hematoma as soon as possible.
